# Scan Range and Radiation Dose of the Ultra-High-Resolution Series in Pancreatic Photon-Counting Detector CT: A Prospective Two-Group Comparison of an Ultra-Low-Dose Planning Series

**DOI:** 10.3390/diagnostics16182926

**Published:** 2026-09-10

**Authors:** Michael A. Arnoldner, Alexander Herold, Andreas Strassl, Klaus Sahora, Veronika Vetchy, Florian Lindenlaub, Dietmar Tamandl

**Affiliations:** 1 Department of Biomedical Imaging and Image-Guided Therapy, Medical University of Vienna, Waehringer Guertel 18-20, 1090 Vienna, Austria; 2 Christian Doppler Laboratory for Machine Learning Driven Precision Medicine, Department of Biomedical Imaging and Image-Guided Therapy, Medical University of Vienna, Waehringer Guertel 18-20, 1090 Vienna, Austria; 3 Department of General Surgery, Division of Visceral Surgery, Medical University of Vienna, Waehringer Guertel 18-20, 1090 Vienna, Austria

**Keywords:** pancreas, tomography, computed, photon-counting detector CT, radiation dosage, radiation protection, scan range, ultra-high resolution

## Abstract

**Background/Objectives**: The ultra-high-resolution (UHR) mode of photon-counting detector computed tomography (PCD-CT) is the most dose-intensive series of a multiphase pancreatic protocol in relation to its scan length, and the pancreas cannot be localised on a topogram. We assessed whether an ultra-low-dose (ULD) unenhanced planning series reduces its scan range and dose without compromising complete depiction of the pancreas. **Methods**: In this prospective single-centre study, 61 pancreatic protocol examinations in 56 patients were performed on a first-generation dual-source PCD-CT. Following a protocol change, the UHR scan range was planned from the topogram in group A (*n* = 30) and from a ULD unenhanced series in group B (n = 31). Dose indices, scan length, margins to the pancreas and completeness of depiction were recorded. **Results**: The UHR scan range was 27% shorter in group B (114 vs. 160 mm; *p* < 0.001), driven by the cranial margin (−58%; *p* < 0.001) rather than the caudal margin (*p* = 0.076). Neither the volume CT dose index nor the size-specific dose estimate of the UHR series differed (*p* = 0.74 and *p* = 0.35), yet its dose–length product fell by 25% (*p* = 0.003) and its share of the dose excluding the mandatory venous phase fell from 62.0% to 50.7% (*p* < 0.001). The total dose was 7.5% lower without reaching significance (*p* = 0.37). Incomplete depiction occurred in 5 of 30 vs. 5 of 31 examinations (*p* = 1.00). The body habitus was closely matched. **Conclusions**: A ULD planning series was associated with a more tightly targeted UHR pancreatic phase, with a 27% shorter scan range and a 25% lower dose–length product for that series, and with no detected increase in incomplete depiction of the pancreas. With topogram-based planning alone, over-scanning was most pronounced at the cranial border. The reduction was confined to the ultra-high-resolution series and was statistically significant there; the total examination dose was lower with ultra-low-dose planning, but this difference was not statistically significant and no conclusion about total dose should be drawn from it.

## 1. Introduction

The ultra-high-resolution (UHR) acquisition mode is one of the genuinely new capabilities of photon-counting detector computed tomography (PCD-CT). The detector is read out at 120 × 0.2 mm collimation, giving a limiting in-plane spatial resolution of approximately 125 μm, far beyond what energy-integrating systems can achieve [1,2]. For the pancreas this matters because the structures that carry diagnostic weight are small: in a series of 20 patients without pancreatic pathology, PCD-CT visualised the ductal system including side ducts, the papillae, the peripancreatic arteries and veins and regional lymph nodes in at least 90% of cases, with moderate to good inter-reader agreement [3], improved the conspicuity of pancreatic ductal adenocarcinoma (PDAC) [4,5], and increased inter-reader agreement for vascular involvement and metastasis detection [6].

This spatial resolution is particularly relevant in pancreatic cancer, where resectability is assessed based on tumour contact with the peripancreatic vessels [7].

The UHR mode is, however, not a standard abdominal acquisition. Its narrow collimation covers only 24 mm of the *z*-axis per rotation against 57.6 mm in the standard mode [1], and it requires an elevated image quality level to preserve the signal-to-noise ratio at the smaller voxel size. In a multiphase pancreatic protocol, the UHR series would represent a substantial contribution to radiation dose, limiting how freely it can be used. Since the dose–length product is, by definition, proportional to the irradiated length, restricting the UHR range to the organ of interest is the most direct way to reduce the dose. Redundant *z*-axis coverage is common: a scoping review of 38 studies reported a mean prevalence of 75% with excess coverage between 12 and about 90 mm [8], and 70.6% of examinations exceeded their predefined anatomic boundaries in a multicentre audit [9].

However, the pancreas cannot be reliably localised on a two-plane topogram, since the technician sees bony landmarks and gas patterns rather than the gland itself. The same scoping review attributes directional over-scanning patterns specifically to scout-image limitations and to radiographer caution about under-coverage [8]. Cross-sectional planning data remove this uncertainty: in coronary CT an ultra-low-dose (ULD) unenhanced planning scan reduced over-scanning without a significant penalty in total dose [10], calcium-score-based planning shortened acquisitions from 134 to 114 mm [11], and low-dose helical localisers or organ prediction on scout views shortened abdominal acquisitions by 26.7 mm and 60 mm, respectively [12,13]. Whether this transfers to the UHR series of a pancreatic PCD-CT protocol has not been studied.

We therefore introduced a ULD unenhanced series, acquired before contrast administration, to delineate the gland and define the borders of the UHR range. The aims of this study were to quantify its effect on the scan range and radiation dose of the UHR series, and to determine whether the tighter range compromised complete depiction of the pancreas.

## 2. Materials and Methods

### 2.1. Study Design and Patients

This study was designed as a prospective, observational single-centre pilot study and was approved by the Ethics Committee of the Medical University of Vienna (protocol code 1920/2022 approved on 16 February 2023); written informed consent was obtained from all the participants. The study was conducted in accordance with the Declaration of Helsinki and is reported in accordance with the STROBE statement. It was not registered in a public trial registry, as it is a purely observational comparison of two clinical protocols and does not prospectively assign participants to an intervention.

**Figure 1:**
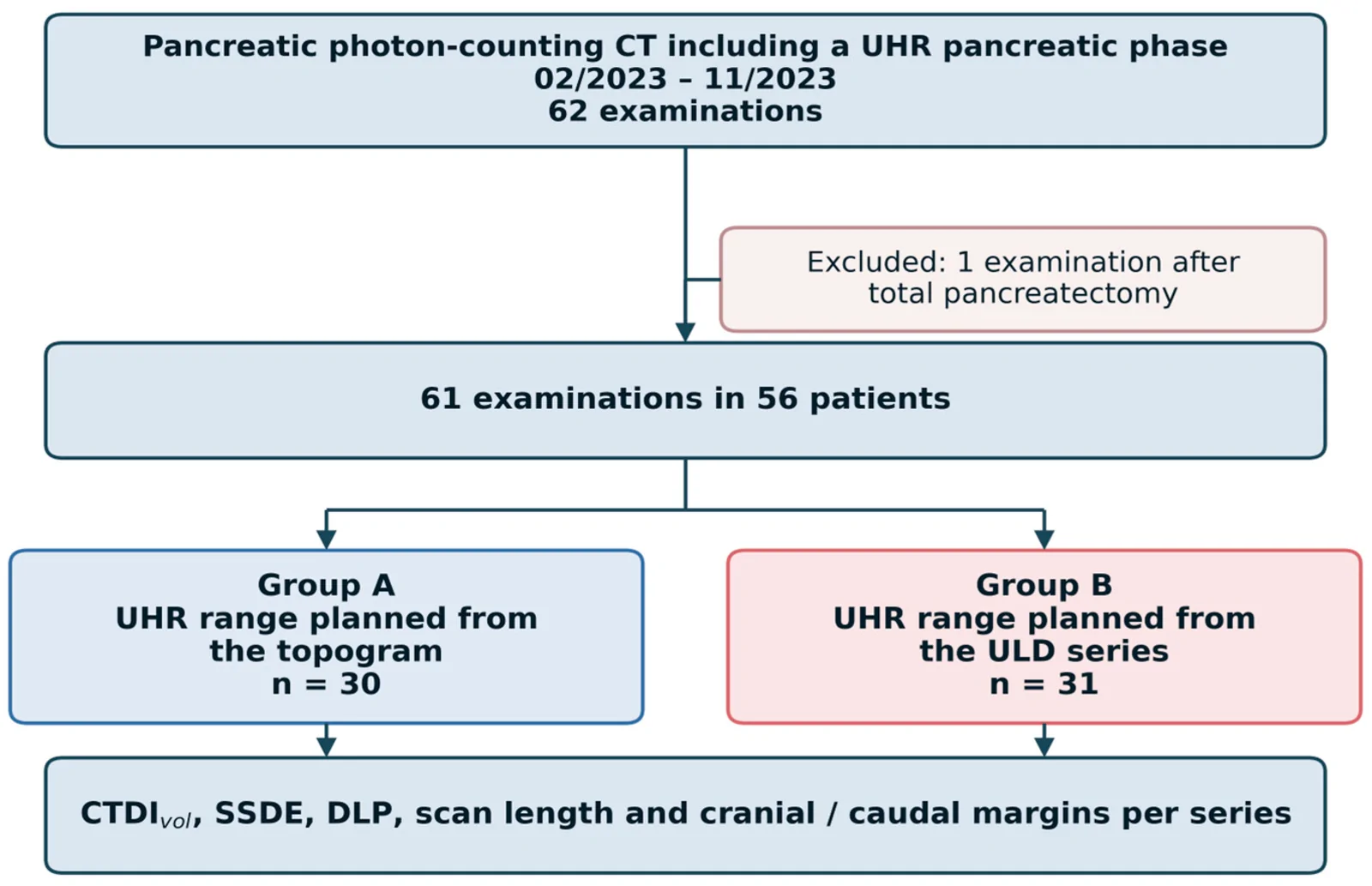
Study flow diagram. Repeat examinations of the same patient were retained, so the unit of analysis is the examination. UHR, ultra-high resolution; ULD, ultra-low dose.

Between February 2023 and November 2023, consecutive patients scheduled for a clinically indicated pancreatic protocol PCD-CT were enrolled, yielding 62 examinations. One examination performed after total pancreatectomy was excluded because in that situation, neither a pancreatic target volume nor a pancreatic reference structure exists, leaving 61 examinations in 56 patients (Figure 1). Four patients underwent more than one examination during the study period, contributing nine examinations; these were retained, so the unit of analysis is the examination rather than the patient. Examinations were performed for suspected or known pancreatic disease, comprising primary diagnosis, staging and follow-up. The underlying pancreatic diagnosis was taken for every examination from the final diagnosis documented in the hospital record. This diagnosis was based on histology or cytology where tissue had been obtained and otherwise on the multidisciplinary tumour board assessment including imaging follow-up; it is therefore not a diagnosis derived from the index CT examination alone. Examinations performed for suspected pancreatic disease in which no pancreatic lesion was found are reported as such.

**Figure 2:**
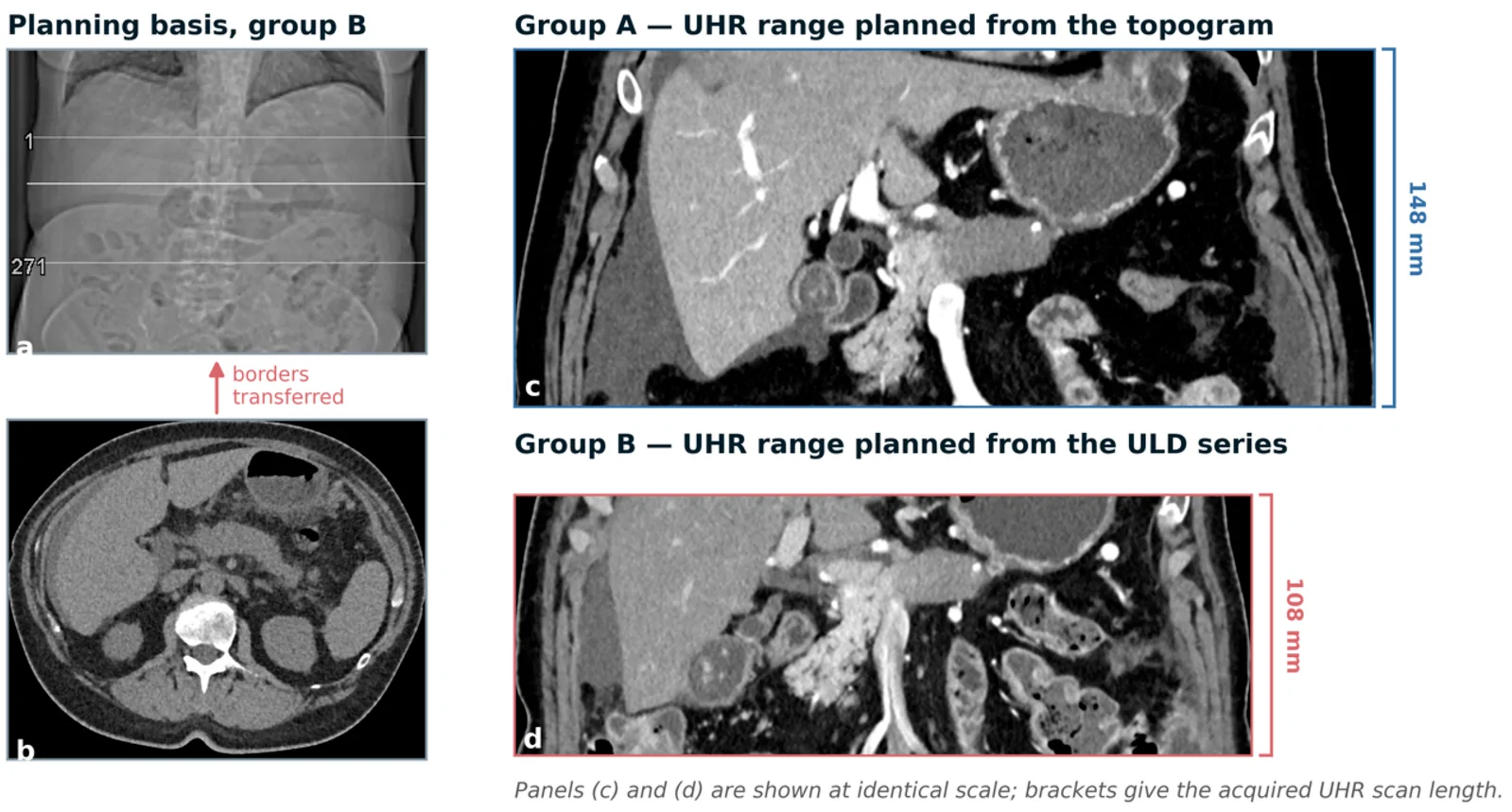
Planning of the ultra-high-resolution range, illustrated in the same 68-year-old woman examined once in each group. (**a**) Topogram of the group B examination with the navigation lines marking the cranial and caudal borders identified on the ultra-low-dose series; while scrolling through the axial images the lines move synchronously on the topogram. (**b**) Axial ultra-low-dose image at the level of the pancreas on which the borders were set. (**c**) Coronal reconstruction of the ultra-high-resolution phase planned from the topogram (scan length 148 mm, cranial margin 51 mm, DLP 341 mGy·cm). (**d**) The same patient five months later, planned from the ultra-low-dose series (108 mm, 17 mm, 208 mGy·cm). Panels (**c**,**d**) are displayed at identical scale, calibrated to the recorded scan lengths. The patient’s weight decreased from 74 to 60 kg between the examinations, which accounts for the smaller body outline in (**d**).

Allocation was not randomised. An anteroposterior and lateral topogram was acquired in every examination of both groups and was used to plan the arterial and venous phases, and in group B, the ULD series was also used; the two groups therefore differ only in how the borders of the UHR pancreatic phase were defined. In group A (n = 30) these borders were set on the topogram itself, as had been standard practice, so that the radiographer had to infer the position of the gland from bony landmarks and gas distribution. In group B (n = 31) an additional ULD unenhanced series of the upper abdomen was acquired before contrast administration, and the radiographer set the borders on these cross-sectional images, on which the gland is directly visible: while scrolling through the ULD series, navigation lines move synchronously on the topogram so that the cranial and caudal borders identified on the axial images are transferred directly to the planning view. No other element of the protocol differed between the groups; an example of both planning approaches is shown in Figure 2. The two groups were consecutive and non-overlapping in time: all group A examinations were performed before the departmental protocol change and group B examinations after it. Assignment therefore followed the calendar period in which the examination took place. All other acquisition and reconstruction parameters were unchanged between the groups. This study was designed as a pilot study, and no formal sample size calculation was performed; the sample was determined by the number of examinations available during the enrolment period.

### 2.2. CT Acquisition

Image acquisition was performed on a first-generation dual-source photon-counting detector CT system (NAEOTOM Alpha, software version VA50; Siemens Healthineers, Forchheim, Germany). The protocol comprised three scan series in group A and four in group B. In group A it included an arterial phase of the upper abdomen, a late arterial (pancreatic) phase of the upper abdomen, and a venous phase covering the entire abdomen and pelvis; when the examination was performed for staging purposes, the venous phase additionally included the thorax. The ULD, arterial and venous series were acquired in QuantumPlus mode, the vendor’s standard multi-energy acquisition mode, with 144 × 0.4 mm collimation, whereas the pancreatic phase was acquired in the ultra-high-resolution mode (HighResUltra) at 120 × 0.2 mm collimation. In UHR mode the CARE keV image quality level was increased from 170 to 250 to preserve the signal-to-noise ratio at the higher spatial resolution, and a reduced slice thickness (0.6 mm vs. 1.0 mm) and a sharper reconstruction kernel (Br48 vs. Br40) were applied. In group B, the ULD series of the upper abdomen was acquired at 100 kVp with tin prefiltration (Sn100), at a CARE keV image quality level of 40—against 170 for the arterial and venous phases—and with an increased slice thickness of 1.5 mm. Rotation time, pitch and quantum iterative reconstruction level were identical across all the series. All the acquisition and reconstruction parameters are given in Table 1.

A contrast agent with an iodine concentration of 400 mg/mL (Iomeron 400; Bracco Imaging, Milan, Italy) was injected with the volume and flow rate adapted to body size (1 mL per kg body weight), resulting in a mean contrast volume of 71 mL and a mean injection rate of 3.3 mL/s.

### 2.3. Dose and Scan Range Evaluation

The volume CT dose index (CTDIvol), dose–length product (DLP) and scan range were recorded for each series from the radiation dose structured report. The water-equivalent diameter and the size-specific dose estimate (SSDE) were reported per series by the scanner according to AAPM Report 220, which defines the water-equivalent diameter and the corresponding conversion factors. CTDIvol and SSDE are per-slice dose indices and are, by definition, independent of the irradiated length; DLP is the corresponding length-integrated quantity. Reporting both therefore separates a change in exposure per slice from a change in coverage. The ratio of the CTDIvol of the UHR series to that of the arterial phase of the same examination was used to describe its relative tube output. The venous phase covers the entire abdomen and pelvis and is a mandatory component of the protocol, so it is not amenable to range optimisation; the DLP of the UHR series is therefore reported both as a share of the total examination DLP and as a share of the optimisable DLP, defined as the total examination DLP minus the venous phase.

To assess the accuracy of scan range planning, over-ranging of the pancreatic phase was quantified by measuring the distance between the pancreatic parenchyma or tumour and the first and the last slice of the acquisition, yielding a cranial and a caudal margin; their sum is reported as the summed margin. A margin of 0 mm denotes that the gland or tumour ended flush with the edge of the acquisition while still being completely covered. Where the gland or tumour extended beyond the edge, no margin exists in that direction, and the value was recorded as missing; these examinations were classified as truncated cranially or caudally. The margins are therefore missing by design rather than being censored, and the margin comparisons are conditional on complete depiction in the respective direction. Each pancreatic phase was rated as depicting the pancreas completely, or as truncated cranially or caudally, by a single reader (A.S., 13 years of experience in abdominal imaging) who was not blinded to group allocation. To assess reproducibility, a second reader (M.A., 12 years of experience in abdominal imaging), blinded to group allocation and to the first reading, independently repeated the assessment of completeness, of the direction of truncation and of both margins in all 61 examinations. Agreement for the categorical assessments was quantified with Cohen’s kappa, and agreement for the margins with the intraclass correlation coefficient for absolute agreement, ICC(2,1), with 95% confidence intervals.

### 2.4. Statistical Analysis

The continuous variables are summarised as median with interquartile range (IQR) and as mean ± standard deviation, and categorical variables as counts with percentages. Because several variables deviated from normality on the Shapiro–Wilk test, non-parametric methods were used throughout. Groups were compared with the Mann–Whitney U test, the Hodges–Lehmann median difference (group B minus group A) and a distribution-free 95% confidence interval (CI) as the effect size, and proportions with Fisher’s exact test and Jeffreys 95% CIs; the absolute difference between two proportions is reported with the Newcombe hybrid score 95% CI.

The DLP of the UHR series was nominated as the primary endpoint and the completeness of pancreatic depiction as the co-primary safety endpoint in the study protocol before enrolment began; all other comparisons are secondary and are reported without adjustment for multiplicity. Because prior partial pancreatic resection was unevenly distributed between the two consecutive groups, the primary endpoint was additionally analysed in a post hoc multivariable linear model on the log-transformed DLP of the UHR series, adjusted for group, prior partial resection, thoracoabdominal extent of the venous phase, sex and age; a second model additionally included body mass index. A post hoc subgroup analysis was restricted to examinations with the pancreas in situ. To account for the retained repeat examinations, all the analyses were repeated on the subset containing one examination per patient. All source values flagged as implausible during a structured data review were verified against the radiation dose structured report and the images and corrected where necessary; the present analysis uses the corrected data set. Group comparisons are reported as medians with interquartile ranges and as Hodges–Lehmann median differences; percentage changes quoted in the text refer to the difference in arithmetic means and are identified as such wherever they are given. Two-sided *p* < 0.05 was considered significant. The analyses used Python 3 (Python Software Foundation, Wilmington, DE, USA) with SciPy 1.16 and NumPy.

## 3. Results

### 3.1. Patients and Examinations

Sixty-one examinations in 56 patients were analysed, 30 in group A and 31 in group B (Figure 1). Age (70 years, IQR 63–76, in group A vs. 68, 56–74, in group B; *p* = 0.35), sex distribution (18/30 vs. 14/31 male; *p* = 0.31), extent of the venous phase (thoracoabdominal in 20/30 vs. 21/31; *p* = 1.00) and prior partial pancreatic resection (6/30 vs. 11/31; *p* = 0.26) did not differ significantly between the groups. Body habitus was closely matched: body mass index was 23.7 kg/m^2^ (21.0–25.5) vs. 24.0 kg/m^2^ (21.2–25.6; *p* = 0.95), and the water-equivalent diameter of the UHR series was 28.8 cm (25.5–30.4) vs. 28.1 cm (25.7–30.4; *p* = 0.99). A pancreatic lesion was present in 48 of 61 examinations (79%), most frequently pancreatic ductal adenocarcinoma (31/61, 51%), followed by neuroendocrine tumour and intraductal papillary mucinous neoplasm (7 each). In the remaining 13 examinations (21%) the study was performed for suspected pancreatic disease, and no pancreatic lesion was identified. The distribution did not differ between the groups (*p* = 0.55 across all categories; 23/30 vs. 25/31 with any lesion, *p* = 0.76; 13/30 vs. 18/31 with adenocarcinoma, *p* = 0.31). The contrast volume was marginally lower in group B (71 mL, IQR 70–80, vs. 70 mL, 60–75; *p* = 0.045) at a comparable flow rate (*p* = 0.13). The characteristics are given in Table 2.

**Table 2:** Patient and examination characteristics.

Characteristic	Group A (n = 30)	Group B (n = 31)	*p*
Age (years)	70 (63–76)	68 (56–74)	0.35
Male sex	18 (60)	14 (45)	0.31
Body mass index (kg/m^2^)	23.7 (21.0–25.5); n = 29	24.0 (21.2–25.6)	0.95
Water-equivalent diameter, UHR series (cm)	28.8 (25.5–30.4); n = 29	28.1 (25.7–30.4)	0.99
Entire pancreas in situ	24 (80)	20 (65)	0.26
Prior partial pancreatic resection	6 (20)	11 (35)	0.26
Venous phase thoracoabdominal	20 (67)	21 (68)	1.00
Underlying diagnosis ^1^			0.55 ^2^
Pancreatic ductal adenocarcinoma	13 (43)	18 (58)	
Neuroendocrine tumour	3 (10)	4 (13)	
Intraductal papillary mucinous neoplasm	5 (17)	2 (6)	
Mucinous cystic neoplasm	0 (0)	1 (3)	
Duodenal carcinoma	1 (3)	0 (0)	
Renal cell carcinoma metastasis	1 (3)	0 (0)	
No pancreatic lesion ^3^	7 (23)	6 (19)	
Any pancreatic lesion	23 (77)	25 (81)	0.76
Contrast volume (mL)	71 (70–80)	70 (60–75); n = 29	0.045
Contrast flow rate (mL/s)	3.5 (3.0–3.8); n = 25	3.0 (3.0–3.5); n = 26	0.13

Unless otherwise indicated, data are numbers of examinations with percentages in parentheses; continuous data are median with interquartile range in parentheses. *p* values from the Mann–Whitney U test or Fisher’s exact test. ^1^ Diagnosis established from the clinical record; where more than one lesion was present, the lesion determining the indication is reported. ^2^ Permutation test across all seven diagnostic categories (20,000 permutations). ^3^ Examination performed for suspected pancreatic disease in which no pancreatic lesion was identified.

### 3.2. Scan Range and Planning Margins

The arterial phase, planned from the topogram in both groups, was of comparable length (215 mm, IQR 198–232, in group A vs. 226 mm, 201–236, in group B; *p* = 0.52). The UHR pancreatic phase was markedly shorter in group B: 160 mm (135–197) vs. 114 mm (99–129), a median difference of −44 mm (95% CI −63 to −24; *p* < 0.001), corresponding to a 27% reduction in the mean scan length. Relative to the arterial phase of the same examination, UHR coverage fell from 0.73 (0.64–0.99) to 0.53 (0.43–0.64; *p* = 0.001).

**Figure 3:**
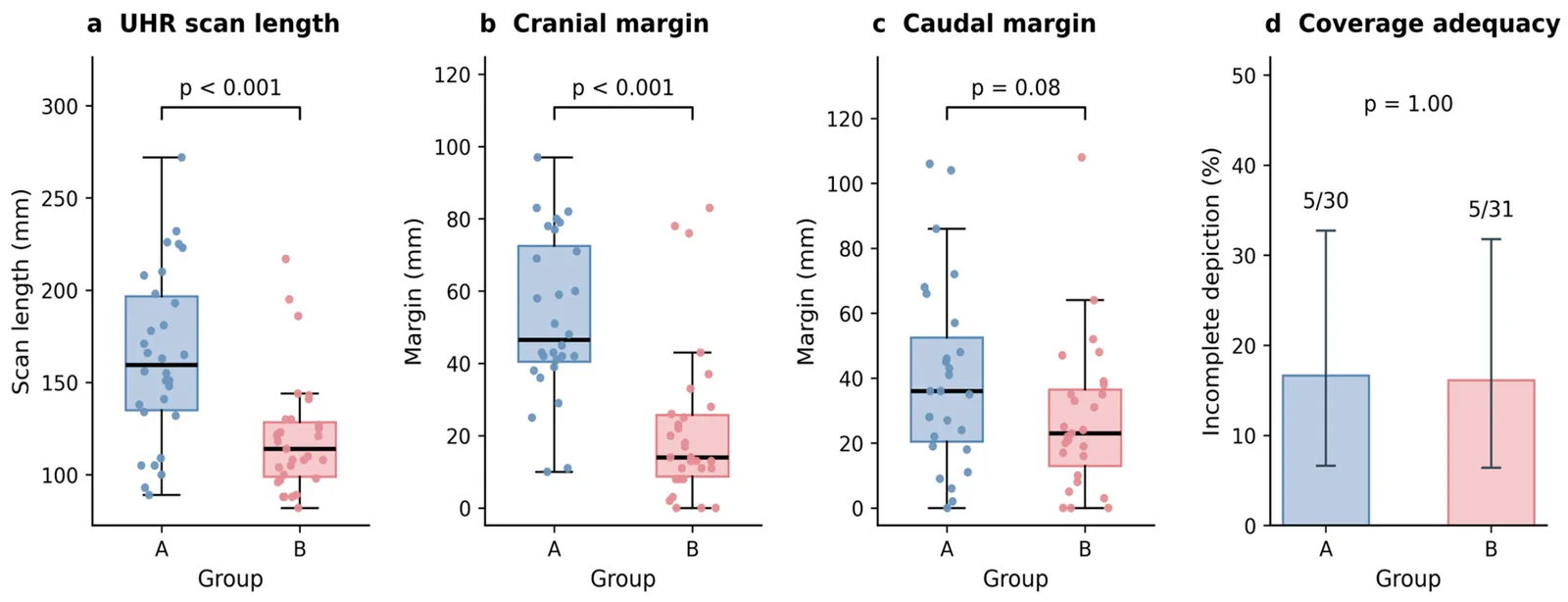
Scan range, planning margins and completeness of depiction. (**a**) Scan length of the ultra-high-resolution (UHR) pancreatic phase. (**b**) Cranial margin between the pancreas and the first slice of the UHR series. (**c**) Caudal margin between the pancreas and the last slice. (**d**) Proportion of examinations with incomplete depiction of the pancreas, with Jeffreys 95% confidence intervals. Boxes show median and interquartile range and whiskers the range excluding outliers; each dot is one examination. A margin of 0 mm denotes a gland ending flush with the edge of the acquisition while still completely covered; where an examination was truncated no margin exists in that direction, so panels (**b**,**c**) are conditional on complete depiction. *p* values from the Mann–Whitney U test (**a**–**c**) and Fisher’s exact test (**d**).

The reduction was almost entirely cranial. The cranial margin fell from 47 mm (41–73) to 14 mm (9–26), a median difference of −32 mm (95% CI −43 to −23; *p* < 0.001) and a 58% reduction in the mean, whereas the caudal margin fell from 36 mm (21–53) to 23 mm (13–37) without reaching significance (−11 mm, −24 to 1; *p* = 0.076). The summed margin fell from 95 mm (77–107) to 35 mm (28–58), a 49% reduction in the mean (−51 mm, −70 to −32; *p* < 0.001). The cranio-caudal extent of the pancreatic target itself, derived as the UHR scan length minus both margins and therefore computable only where the gland was completely covered, did not differ between the groups: 74 mm (58–97; n = 25) vs. 76 mm (55–96; n = 26), a median difference of 0 mm (95% CI −16 to 17; *p* = 0.99). Prior partial resection shortened the target as expected (83 mm vs. 52 mm; *p* < 0.001), but this effect was distributed similarly across the two groups. Margin comparisons are conditional on complete depiction in the respective direction and are therefore based on 28 and 30 examinations cranially and on 27 and 27 caudally. The distributions are shown in Figure 3a–c.

### 3.3. Completeness of Pancreatic Depiction

The pancreas was incompletely depicted by the UHR series in 5/30 examinations in group A (16.7%, 95% CI 6.7–32.7) and in 5/31 in group B (16.1%, 6.4–31.8; *p* = 1.00). Truncation was predominantly caudal in both groups (group A two cranial and three caudal, group B one cranial and four caudal). The absolute difference between the groups was −0.5 percentage points (95% CI −19.7 to +18.4, Newcombe method). Despite a 27% shorter acquisition and a 58% smaller cranial margin, no increase in the proportion of examinations with incomplete depiction was detected (Figure 3d). The width of this interval should be noted: with approximately 30 examinations per group, and without a sample size calculation for this endpoint, the data are compatible with a clinically relevant increase as well as with a decrease, and the comparison does not establish equivalence or non-inferiority. The second blinded reader reached the same conclusion in all 61 examinations, both for completeness (agreement 61/61, Cohen’s kappa 1.00) and for the direction of truncation (61/61, kappa 1.00). Agreement for the measured margins was likewise near-perfect (cranial ICC(2,1) 1.00, 95% CI 0.999–1.000, n = 58; caudal 1.00, 0.999–1.000, n = 54), with a mean difference of −0.1 mm and −0.3 mm, respectively, and no individual deviation exceeding 2 mm. The reduction was achieved at the cranial border while the residual truncations remained predominantly caudal, so the shortening did not act in the direction in which coverage was lost.

### 3.4. Radiation Dose

**Figure 4:**
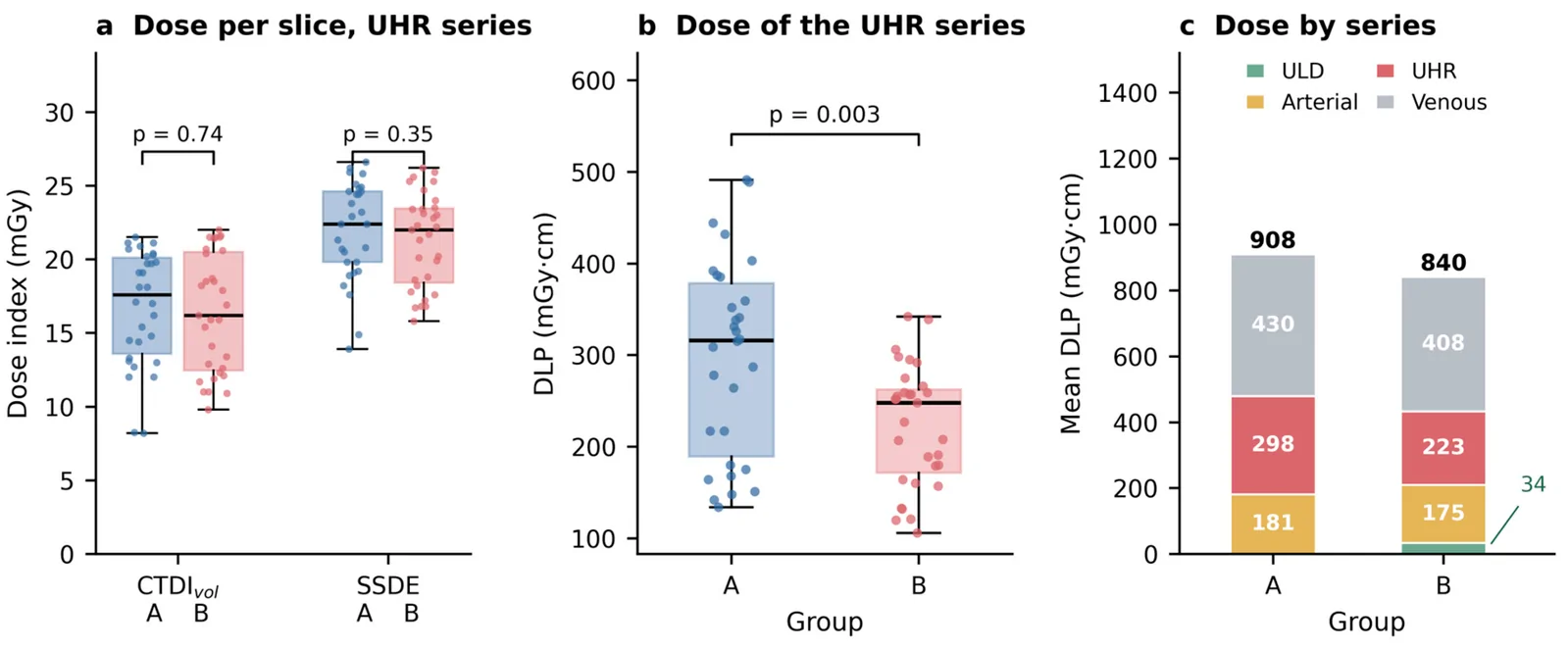
Radiation dose. (**a**) Volume CT dose index by series; the ultra-high-resolution (UHR) series is acquired at about 2.4 times the tube output of the arterial phase, and the tube output did not differ between the groups. The *p* value shown compares the UHR series between the groups; comparisons for the arterial and ultra-low-dose (ULD) series are given in Table 3. (**b**) Dose–length product of the UHR series. (**c**) Mean dose–length product by series; values are arithmetic means, whereas all the other dose data in this article are medians. The dose saved on the UHR series is partly offset by the ULD planning series, and the venous phase, which covers the abdomen and pelvis and is a mandatory part of the protocol, remains the largest single contributor in both groups.

The UHR series was the most dose-intensive acquisition of the protocol in relation to its scan length. Its CTDIvol was about 2.4 times that of the arterial phase of the same examination, a relationship that was identical in both groups (2.37, IQR 2.30–2.45, vs. 2.42, 2.31–2.48; *p* = 0.31), and per centimetre of irradiated length it delivered 19.3 mGy·cm (14.6–22.4) against 8.2 mGy·cm (6.4–10.1) for the arterial phase. Neither per-slice dose index of the UHR series differed between the groups: CTDIvol was 17.6 mGy (13.6–20.1) vs. 16.2 mGy (12.5–20.5; *p* = 0.74) and SSDE 22.4 mGy (19.8–24.6) vs. 22.0 mGy (18.4–23.5; *p* = 0.35). The same held true for the arterial phase (CTDIvol *p* = 0.52, SSDE *p* = 0.39; Figure 4a). Because SSDE corrects the exposure index for patient size, its equality confirms that neither the tube output nor the body habitus of the two groups differed.

**Table 3:** Scan range, planning margins and radiation dose.

Parameter	Group A (n = 30)	Group B (n = 31)	Δ (95% CI)	*p*
Scan length, arterial phase (mm)	215 (198–232); 214.6 ± 27.5; n = 30	226 (201–236); 212.4 ± 40.9; n = 30	5 (−11 to 19)	0.52
Scan length, UHR series (mm)	160 (135–197); 163.9 ± 46.4; n = 30	114 (99–129); 120.2 ± 31.5; n = 31	−44 (−63 to −24)	<0.001
Relative UHR coverage	0.73 (0.64–0.99); n = 30	0.53 (0.43–0.64); n = 30	−0.19 (−0.31 to −0.07)	0.001
Cranial margin, UHR series (mm)	47 (41–73); 52.8 ± 22.3; n = 28	14 (9–26); 21.9 ± 22.1; n = 30	−32 (−43 to −23)	<0.001
Caudal margin, UHR series (mm)	36 (21–53); 40.1 ± 28.8; n = 27	23 (13–37); 27.5 ± 23.3; n = 27	−11 (−24 to 1)	0.076
Summed margin, UHR series (mm)	95 (77–107); 95.9 ± 33.9; n = 25	35 (28–58); 48.8 ± 40.5; n = 26	−51 (−70 to −32)	<0.001
CTDIvol, ULD series (mGy)	not acquired	1.6 (1.3–2.0); 1.60 ± 0.60; n = 31	—	—
CTDIvol, arterial phase (mGy)	7.2 (5.6–8.9); 7.18 ± 1.89; n = 30	6.8 (5.4–8.1); 7.02 ± 2.07; n = 31	−0.3 (−1.4 to 0.7)	0.52
CTDIvol, UHR series (mGy)	17.6 (13.6–20.1); 16.72 ± 3.88; n = 30	16.2 (12.5–20.5); 16.35 ± 4.02; n = 31	−0.4 (−2.5 to 1.5)	0.74
CTDIvol ratio, UHR/arterial	2.37 (2.30–2.45); n = 30	2.42 (2.31–2.48); n = 31	0.03 (−0.03 to 0.09)	0.31
SSDE, ULD series (mGy)	not acquired	2.2 (1.9–2.5); 2.11 ± 0.63; n = 31	—	—
SSDE, arterial phase (mGy)	10.1 (8.2–11.3); 9.92 ± 1.89; n = 29	9.5 (8.3–10.9); 9.57 ± 1.82; n = 31	−0.5 (−1.4 to 0.5)	0.39
SSDE, UHR series (mGy)	22.4 (19.8–24.6); 21.96 ± 3.36; n = 29	22.0 (18.4–23.5); 21.30 ± 3.17; n = 31	−0.9 (−2.5 to 1.0)	0.35
DLP, ULD series (mGy·cm)	not acquired	32 (26–42); 33.7 ± 14.8; n = 31	—	—
DLP, arterial phase (mGy·cm)	173 (144–221); 181.1 ± 54.5; n = 30	166 (129–214); 175.2 ± 62.0; n = 31	−9 (−40 to 23)	0.47
DLP, UHR series (mGy·cm)	316 (189–379); 297.9 ± 107.5; n = 30	248 (172–263); 223.4 ± 66.4; n = 31	−74 (−130 to −26)	0.003
DLP, venous phase (mGy·cm)	394 (355–498); 429.6 ± 140.3; n = 30	391 (314–530); 407.9 ± 122.9; n = 31	−16 (−70 to 49)	0.65
DLP, optimisable (total − venous) (mGy·cm)	488 (351–590); 478.9 ± 155.0; n = 30	421 (351–532); 432.3 ± 125.1; n = 31	−46 (−116 to 26)	0.28
DLP, total examination (mGy·cm)	897 (743–1140); 908.5 ± 245.4; n = 30	796 (644–1039); 840.2 ± 236.9; n = 31	−67 (−192 to 66)	0.37
UHR series as share of optimisable DLP (%)	62.0 (58.3–67.1); 61.5 ± 6.0; n = 30	50.7 (47.1–55.9); 51.9 ± 7.4; n = 31	−10.5 (−13.8 to −6.5)	<0.001
UHR series as share of total DLP (%)	33.4 (27.9–36.6); 32.5 ± 7.0; n = 30	26.5 (23.3–29.4); 26.9 ± 5.2; n = 31	−6.1 (−9.4 to −2.5)	0.001
Venous phase as share of total DLP (%)	47.1 (41.8–53.3); 47.6 ± 8.2; n = 30	47.8 (45.4–51.1); 48.4 ± 4.6; n = 31	1.4 (−2.1 to 4.7)	0.42
Incomplete depiction of the pancreas	5 (16.7)	5 (16.1)	—	1.00 ^1^

Data are median (interquartile range); mean ± standard deviation; and number of observations, except for incomplete depiction, which is the number of examinations with the percentage in parentheses. Δ is the Hodges–Lehmann median difference (group B minus group A) with a 95% confidence interval; *p* values from the Mann–Whitney U test unless indicated otherwise. ^1^ Fisher’s exact test. CI, confidence interval; CTDIvol, volume CT dose index; DLP, dose–length product; UHR, ultra-high resolution; ULD, ultra-low dose.

The DLP of the UHR series nevertheless fell from 316 mGy·cm (189–379) to 248 mGy·cm (172–263), a median difference of −74 mGy·cm (95% CI −130 to −26; *p* = 0.003) and a 25% reduction in the mean (Figure 4b). Pooled across the groups, the DLP of the UHR series scaled with its scan length (Spearman ρ = 0.74; *p* < 0.001; R^2^ = 0.53). The saving is therefore geometric: with the dose per slice and the body habitus unchanged, the entire difference is based on the shorter irradiated length. In the post hoc multivariable model, group B remained associated with a 17.2% lower DLP of the UHR series (95% CI −30.6 to −1.2%; *p* = 0.037), and prior partial resection with a 25.2% lower DLP (−38.7 to −8.9%; *p* = 0.005). Adding body mass index, which was itself strongly associated with the DLP of the UHR series (+5.9% per unit, 95% CI +3.3 to +8.5%; *p* < 0.001), raised the model R^2^ from 0.31 to 0.51 and left group B associated with an 18.8% lower DLP (−30.4 to −5.4%; *p* = 0.008), while the effect of prior resection was attenuated to −15.2% (−29.0 to +1.2%; *p* = 0.066).

The ULD planning series delivered a CTDIvol of 1.6 mGy (1.3–2.0), an SSDE of 2.2 mGy (1.9–2.5) and a DLP of 32 mGy·cm (26–42), approximately one-tenth of the per-slice dose of the UHR series. Averaged over the examinations, 74 mGy·cm were saved on the UHR series and 34 mGy·cm were added by the planning series, so that 45% of the gross saving was consumed by the planning acquisition and a net reduction of 41 mGy·cm remained, corresponding to 14% of the UHR dose of group A.

The venous phase accounted for 47.1% (41.8–53.3) and 47.8% (45.4–51.1) of the total examination DLP in groups A and B (*p* = 0.42) and was the largest single contributor in both (394 mGy·cm, 355–498, vs. 391 mGy·cm, 314–530; *p* = 0.65; Figure 4c). Referred to the optimisable DLP, the share attributable to the UHR series fell from 62.0% (58.3–67.1) to 50.7% (47.1–55.9), a median difference of −10.5 percentage points (95% CI −13.8 to −6.5; *p* < 0.001). Referred to the whole examination, the corresponding shares were 33.4% (27.9–36.6) and 26.5% (23.3–29.4; *p* = 0.001). The total examination DLP was 7.5% lower in group B in the mean (897 mGy·cm, 743–1140, vs. 796 mGy·cm, 644–1039), and the optimisable DLP was 9.7% lower (488 mGy·cm, 351–590, vs. 421 mGy·cm, 351–532), but neither difference reached significance (−67 mGy·cm, 95% CI −192 to +66, *p* = 0.37; and −46 mGy·cm, −116 to +26, *p* = 0.28). Complete dose and scan range data are given in Table 3.

### 3.5. Subgroup Without Prior Partial Resection

In the 44 examinations in which the entire pancreas was in situ, the group difference persisted for every coverage and dose endpoint and was numerically larger than in the whole cohort, while CTDIvol and SSDE remained equal between the groups (Table 4). Incomplete depiction occurred in 5/24 and 3/20 examinations (*p* = 0.71).

### 3.6. Sensitivity Analysis

Restricting the analysis to one examination per patient (26 per group) gave concordant results: UHR scan length 160 vs. 112 mm (*p* < 0.001), DLP of the UHR series 312 vs. 217 mGy·cm (*p* = 0.005), unchanged CTDIvol (*p* = 0.64) and SSDE (*p* = 0.33), cranial margin 44 vs. 14 mm (*p* < 0.001), summed margin 90 vs. 33 mm (*p* < 0.001), UHR share of the optimisable DLP 62.0% vs. 50.9% (*p* < 0.001), unchanged total DLP (*p* = 0.29), unchanged proportion of incomplete depiction (3/26 vs. 4/26; *p* = 1.00), and a group effect of −18.9% (95% CI −33.6 to −0.9%; *p* = 0.041) in the multivariable model.

## 4. Discussion

ULD-based planning of the UHR pancreatic phase was associated with a 27% shorter scan range (*p* < 0.001) and a 25% lower dose–length product of that series (*p* = 0.003), with no detected increase in incomplete depiction of the pancreas (*p* = 1.00). Referring to the part of the protocol that can be shortened at all, the UHR series fell from 62.0% to 50.7% of the delivered dose (*p* < 0.001).

The body mass index and water-equivalent diameter were nearly identical between the groups, neither the CTDIvol nor the SSDE of the UHR series differed, and the arterial phase was of unchanged length; at the same time the dose of the UHR series scaled with its scan length across the pooled cohort (ρ = 0.74). Because SSDE is a per-slice index corrected for patient size, its equality establishes that neither the exposure per slice nor the body habitus contributed, leaving the shorter irradiated length as the only source of the difference. The reduction is located almost entirely at the cranial border: the cranial margin fell by 58% in the mean, whereas the caudal margin fell by 31% and did not reach significance. In body CT generally, over-scanning is more frequent caudally than cranially [14], but the cranial extent of the pancreas is precisely what a topogram cannot show, and the radiographer compensates with a superior safety margin—a behaviour that the over-scanning literature attributes directly to scout-image limitations and cautions about under-coverage [8]. The magnitude is consistent with the analogous literature, in which cross-sectional or automated planning shortened abdominal acquisitions by medians of 26.7 mm and 60 mm [12,13] and coronary acquisitions from 127 to 105 mm [15] and from 134 to 114 mm [11].

For the examination as a whole, only a non-significant trend remained: total DLP was 7.5% lower in group B (*p* = 0.37). Two factors explain this. First, the ULD planning series itself delivers a radiation dose, which partly offsets the reduction achieved in the UHR series. Second, the venous phase is not a candidate for range optimisation at all. It covers the entire abdomen and pelvis, as well as the thorax when staging is requested, and it accounted for roughly 47% of the total dose in both groups. Any range-based intervention on the UHR series is therefore diluted by a fixed component that is half of the examination, which is why the share of the optimisable dose is the more meaningful denominator: on that measure the effect is large and significant. It also identifies where the remaining potential lies. Registry analyses of nearly one million abdominal examinations identify the number of non-indicated phases, rather than the exposure per phase, as the dominant driver of excess abdominal CT dose [16], so a protocol in which the venous phase is half the dose is unlikely to be optimised further by geometry alone.

The interpretation of the mechanism also needs care, because the radiographer, not an automated algorithm, translates the planning image into a scan range. Two aspects deserve emphasis. First, the pancreas is not always easy to identify on the ULD series, particularly in slim patients in whom the peripancreatic fat planes that separate the gland from adjacent structures are thin. Second, the radiographers received no dedicated training run before the protocol change and are used to defining abdominal scan ranges on the topogram rather than on cross-sectional images; the effect reported here is therefore achieved without a learning phase and may underestimate what is achievable with structured training or with automated organ detection [13]. At the same time, the design cannot separate the contribution of the image itself from that of a protocol whose explicit purpose was to shorten the UHR range; both changed simultaneously.

Importantly, shorter UHR coverage was not associated with a higher rate of incomplete pancreatic depiction. Incomplete depiction of the pancreas was similarly frequent in the two groups, with an absolute difference of −0.5 percentage points (95% CI −19.7 to +18.4), and the two readers agreed on every examination. This interval is wide, and the observation should be read as the absence of a detected difference rather than as evidence that the two approaches are equally safe; this study was not powered for this endpoint and cannot exclude a clinically relevant increase. Incomplete depiction by the UHR series does not, however, mean that part of the pancreas was absent from the examination: by protocol the venous phase covers the entire abdomen and pelvis, and the arterial phase covers the upper abdomen so that both include the whole pancreas. The gland was therefore completely imaged on at least two further phases in every case, and no examination had to be repeated. What is lost is the additional spatial resolution of the UHR mode over part of the gland, not the anatomical information. The direction of the residual truncations explains why. They were predominantly caudal in both groups, whereas the shortening acted almost entirely at the cranial border, so the intervention did not encroach on the border at which coverage was actually lost. Any further gain in coverage would therefore have to come from caudal planning rather than from refinement of the cranial margin.

Whether a patient is offered upfront surgery follows from the resectability category assigned at CT, and that category is decided on the degree of contact between the tumour and the peripancreatic vessels: in 368 consecutive patients undergoing upfront resection, margin-negative resection was achieved in 81% of tumours classified as resectable, in 67% of borderline resectable and in 11% of unresectable tumours [7]. Higher spatial resolution improves the depiction of that interface: a high-resolution pancreatic protocol with thinner sections raised the contrast-to-noise ratio of pancreatic ductal adenocarcinoma from 7.1 to 10.4 against a standard protocol, although the prediction of a margin-negative resection did not differ significantly [17]. A dedicated protocol is also not what many patients arrive with: in a registry review of 131 patients staged for this tumour, only 52.7% had been examined with a dedicated pancreatic protocol, and subspecialist re-reading changed the stage in almost one in five cases [18]. Because the tumour is frequently first suspected on an examination performed for another indication, a dedicated ultra-high-resolution study might be requested afterwards for surgical planning, so the targeted acquisition is added to imaging the patient has already received. In that situation the range of the UHR series is the part of the added examination that can be controlled without touching image quality: the present data show that it can be shortened by more than a quarter.

### Limitations

This study has several limitations. It was single-centre, and the two groups were consecutive rather than randomised, so operator learning and case mix could have contributed; the unchanged arterial scan length, per-slice dose indices and body habitus argue against a large effect but do not exclude one. Prior partial pancreatic resection was more frequent in group B and was associated with a lower dose; adjustment for body mass index attenuated this effect while strengthening the group effect, but residual confounding cannot be excluded. Repeat examinations of four patients were retained, so the observations are not fully independent; the analysis restricted to one examination per patient reproduced the primary result. The margins are undefined in the direction in which an examination was truncated and are therefore missing by design, so the margin comparisons are conditional on complete depiction and describe only those examinations in which the planning succeeded. Completeness of depiction was assessed by an unblinded first reader, although a blinded second reader repeated the assessment in all the examinations with complete agreement. We recorded only whether the gland was completely covered and in which direction it was truncated; the extent of the missing portion and whether a pancreatic lesion or a relevant peripancreatic structure lay outside the UHR range, even though it was captured on the venous and arterial phases, were not recorded, so the diagnostic consequence of the residual truncations cannot be quantified from these data. No examination had to be repeated. Because the groups were consecutive rather than randomised, a learning effect among the radiographers, who became more aware of the scan range over the study period, cannot be excluded as a contributor to the observed difference. No qualitative image reading or assessment of diagnostic accuracy was performed, so we cannot state whether the shorter range ever excluded a clinically relevant finding beyond the gland itself.

## 5. Conclusions

Planning the ultra-high-resolution pancreatic phase of photon-counting detector CT on an ultra-low-dose unenhanced series was associated with a more tightly targeted acquisition, with a 27% shorter scan range and a 25% lower dose–length product for that series, and with no detected increase in incomplete depiction of the pancreas. Because the two groups were consecutive rather than randomised, a learning effect among the radiographers cannot be excluded as a contributor. With topogram-based planning alone, over-scanning was most pronounced at the cranial border. The total examination dose was lower with ultra-low-dose planning, but the difference did not reach statistical significance.

## Figures and Tables

**Table 1:** Acquisition and reconstruction parameters.

Parameter	ULD Series (Group B)	Arterial Phase	Pancreatic Phase (UHR)	Venous Phase
Scan mode	QuantumPlus	QuantumPlus	HighResUltra	QuantumPlus
Collimation (mm)	144 × 0.4	144 × 0.4	120 × 0.2	144 × 0.4
Tube voltage (kVp)	Sn100	120	120	120
Spectral shaping	Tin filter	None	None	None
CARE keV image quality level	40	170	250	170
Rotation time (s)	0.5	0.5	0.5	0.5
Pitch	0.8	0.8	0.8	0.8
Start delay after contrast injection (s)	-	15	35	70
Slice thickness (mm)	1.5	1.0	0.6	1.0
Increment (mm)	1.0	0.8	0.4	0.8
Reconstruction kernel	Br40	Br40	Br48	Br40
Matrix size	Automatic	Automatic	Automatic	Automatic
Quantum iterative reconstruction level	3	3	3	3
Anatomic coverage	Upper abdomen	Upper abdomen	Pancreas	Abdomen and pelvis ± thorax

The ULD series precedes contrast administration and is a genuinely unenhanced acquisition; it was acquired 4 s after the breath-hold command and therefore has no delay relative to contrast injection. Sn100 denotes 100 kVp with tin prefiltration. UHR, ultra-high resolution; ULD, ultra-low dose.

**Table 4:** Post hoc subgroup analysis restricted to examinations with the pancreas in situ (n = 44).

Parameter	Group A	Group B	Δ (95% CI)	*p*
Scan length, UHR series (mm)	169 (146–209); n = 24	121 (107–133); n = 20	−48 (−74 to −26)	<0.001
Relative UHR coverage	0.77 (0.68–1.00); n = 24	0.55 (0.46–0.64); n = 19	−0.24 (−0.37 to −0.10)	<0.001
Cranial margin (mm)	55 (42–76); n = 22	14 (10–28); n = 20	−35 (−48 to −25)	<0.001
Summed margin (mm)	97 (81–128); n = 19	31 (21–46); n = 17	−66 (−86 to −44)	<0.001
CTDIvol, UHR series (mGy)	17.6 (14.1–20.2); n = 24	18.0 (13.1–20.9); n = 20	0.2 (−2.3 to 2.2)	0.84
SSDE, UHR series (mGy)	22.4 (20.2–24.6); n = 23	22.6 (19.6–23.5); n = 20	−0.6 (−2.3 to 1.5)	0.55
DLP, UHR series (mGy·cm)	332 (252–388); n = 24	256 (203–279); n = 20	−81 (−135 to −22)	0.007
UHR series as share of total DLP (%)	34.5 (31.1–38.2); n = 24	27.4 (24.5–30.4); n = 20	−6.8 (−10.2 to −3.1)	0.002
Incomplete depiction of the pancreas	5/24 (20.8)	3/20 (15.0)	—	0.71 ^1^

Data are median (interquartile range) and number of observations. Δ is the Hodges–Lehmann median difference (group B minus group A) with a 95% confidence interval; *p* values from the Mann–Whitney U test unless indicated otherwise. ^1^ Fisher’s exact test. Abbreviations as in Table 3.

## Data Availability

The data presented in this study are available on request from the corresponding author due to privacy restrictions on patient imaging and dosimetry records.
